# Comparison of the posterior teeth angulations in orthodontic patients with different facial growth patterns

**DOI:** 10.4317/jced.60657

**Published:** 2023-08-01

**Authors:** Oscar Ledesma-Peraza, Marco Sánchez-Tito

**Affiliations:** 1 Facultad de Ciencias de la Salud, Universidad Privada de Tacna, Tacna, Peru; 2 Specialist in Orthodontics and Maxillary Orthopedics; MSc of Scientific Research and Innovation, PhD in Stomatology

## Abstract

**Background:**

Dental relationships can be affected according to the pattern of facial growth. Thus, the aim of this study was to compare the angulations of posterior teeth in patients with different patterns of facial growth.

**Material and Methods:**

A total of 412 lateral head radiographs were included. The facial skeletal growth pattern was evaluated with the Björk-Jarabak analysis. For the angular measurements of the posterior teeth, the apex-cusp inclination of the premolars and the long axes of the molars were considered, with respect to the occlusal, palatal and mandibular plane. The intraclass correlation coefficient (ICC) was used to evaluate the intra-examiner concordance. One way ANOVA was used to compare the values between the patterns of facial growth. A significance level was set at 0.05.

**Results:**

A high intra-examiner correlation was observed (0.75). Subjects with horizontal and normal growth presented significantly different angulations for the first and second premolars in relation to the palatal plane than the vertical growth (*p*<0.05). Subjects with horizontal growth pattern showed greater angulation of the teeth with respect to the occlusal plane than the other groups (*p*<0.05). The angulations of all mandibular teeth related to the mandibular plane were significantly higher for the group with horizontal growth (*p*<0.05).

**Conclusions:**

The first and second premolars presented greater angulations in subjects with horizontal and normal growth than in those with vertical growth. The angulations of all mandibular teeth were significantly higher in the horizontal growth according to the mandibular plane.

** Key words:**Facial growth pattern, posterior tooth, occlusal plane, palatal plane, mandibular plane.

## Introduction

Malocclusions are the result of the imbalance between various factors such as the pattern of facial growth, oral dysfunction, environmental disturbances, inter- and intra-arch alveolar-dental discrepancies, among others (1-5). Treatment planning must consider the etiological factors of malocclusion, as well as aspects related to clinical and demographic conditions in order to establish the best orthodontic treatment planning (6,7).

Skeletal growth patterns can be expressed in the facial characteristics of patients, influencing even the stages of skeletal maturation (6,8,9). A vertical facial growth pattern is related to an increase in the facial height of the lower third of the face, as well as a tendency for mandibular rotation in a clockwise direction, which may also affect the relationships of the positions of the teeth with respect to their alveolar bases, especially the mesiodistal angulations and their verticalization with respect to reference planes such as the palatal and mandibular planes (10). In addition, the vertical facial growth patterns is associated with compressed arches, posterior crossbites and a greater possibility of developing anterior open bites (10-12). On the other hand, horizontal growth patterns are usually associated with decreased facial height and a tendency to a counterclockwise rotation of the mandible; and at the dentoalveolar level, it is common to observe the presence of deep bites (11).

As mentioned earlier, dental relationships can be affected according to the pattern of facial growth. In this regard, Kim reported that in subjects with a vertical growth pattern and who developed open bites, the posterior teeth presented a mesial angulation in relation to the occlusal plane (13). This characteristic may be associated with the fact that mandibular growth trend have been shown to affect tooth eruption patterns (14). In this sense, Tsai found that in patients with vertical growth patterns, the incisors were more vertical (15).

Reference planes such as the palatal plane, mandibular plane, and occlusal plane have been widely described when establishing craniofacial growth characteristics (16-19), although it is known that these references can undergo modifications throughout growth and as part of mainly orthopedic mechanics.

The literature is limited in studies that relate the positions of the teeth with respect to facial growth trends, it is important to distinguish the different patterns in order to establish treatment plans that allow sTable results considering the variabilities and individual characteristics of the patients. Badiee *et al*., evaluated the mesiodistal angulations of the posterior teeth in the various facial patterns, their results showed that a greater forward inclination of the posterior teeth was observed in the vertical pattern (20). Janson *et al*., compared the angulations of posterior teeth in patients with normal occlusion and open bite, finding that the maxillary and mandibular premolars were more angulated mesially with respect to the occlusal plane (21).

Therefore, the aim of this study was to compare the angulations of posterior teeth in patients requiring orthodontic treatment with different patterns of facial growth.

## Material and Methods

This retrospective cross-sectional study was approved by the ethics committee of the Faculty of Health Sciences, Universidad Privada de Tacna, Tacna, Peru, with research protocol number 85/FACSA/UI.

The sample consisted of 412 lateral head radiographs of subjects without previous orthodontic treatment and with complete dentition up to the second molars. The radiographs were obtained from the records of five orthodontic offices in the city of Tacna, Peru. All radiographs were taken by the same radiographic center, using the Orthophos SL 3D Ceph radiographic system (Dentsply Sirona, Germany), operated at 85 kVp and 8 mA, with an exposure time of 14.18 s and a Voxel size of 80 µm. Cephalometric tracing and identification of landmarks were performed by hand in a dark room by the same investigator (O.L.P.) to reduce inter-examiner differences. A negatoscope, cephalometric paper (GAC International Inc, Bohemia, NY, USA) and a 0.5 mm 3H graphite pencil (A. W. Faber-Castell, Stein, Germany) were used. A total of eight traces were made per day, to avoid visual fatigue of the operator.

The facial skeletal growth pattern was evaluated with the relationship between the angular measurements of the Björk-Jarabak analysis (22). The total sum of the sella turcica, articular and gonial angles, was considered. Values of 396°±6° represent normal growth (mesofacial), decreased values indicate horizontal growth (brachyfacial), while increased values indicate vertical growth (dolichofacial).

**Figure 1:**
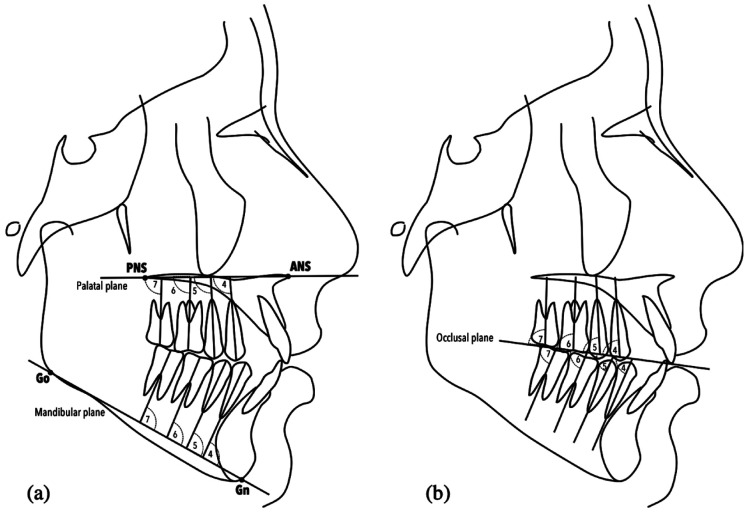
Reference planes. (a) Posterior maxillary and mandibular teeth in relation to palatal and mandibular planes. (b) Posterior maxillary and mandibular teeth in relation to the occlusal plane.

For the angular measurements of the posterior teeth, the apex-cusp inclination of the premolars and the long axes of the molars were considered, with respect to the occlusal plane (OP), the palatal plane (ANS-PNS) and the mandibular plane (Go-Gn), according to a previous study (20). The description of each of the angular measurements is detailed in Table 1. A representation of the measurements can be seen in Figure 1.

**Table 1:**
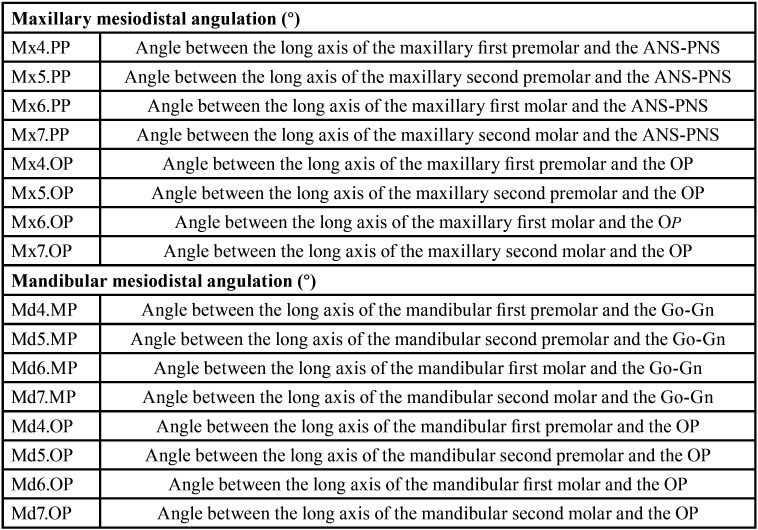
Description of the cephalometric magnitudes.

To assess intra-examiner error, 20% of the sample was randomly selected. Tracings and measurements were made by the same investigator with an interval of two weeks.

-Statistical analysis

For the intra-examiner evaluation, the intraclass correlation coefficient (ICC) was used, which allows measuring the level of concordance that exists between two or more assessments of a quantitative nature, considering a confidence level of 95% and a significance level of 5%. Normal distribution of the data was evaluated with the Shapiro-Francia test. All quantitative data had a normal distribution. The comparison of all the values of the measurements between the patterns of facial growth was carried out with the one-way ANOVA test followed by the Bonferroni post hoc test. All statistical analyses were performed with the Stata® 17 Software (StataCorp LP, College Station, TX, USA) for MacOS. The significance level considered in this study was 5%.

## Results

The interclass correlation coefficient test showed coefficients with values greater than 0.75, with a 95% confidence interval for all variables. This indicates a high correlation between intra-examiner measurements.

Table 2 shows the characteristics of the subjects included in the sample of radiographs. A predominance of the normal growth pattern is observed, over vertical and horizontal growth.

**Table 2:**
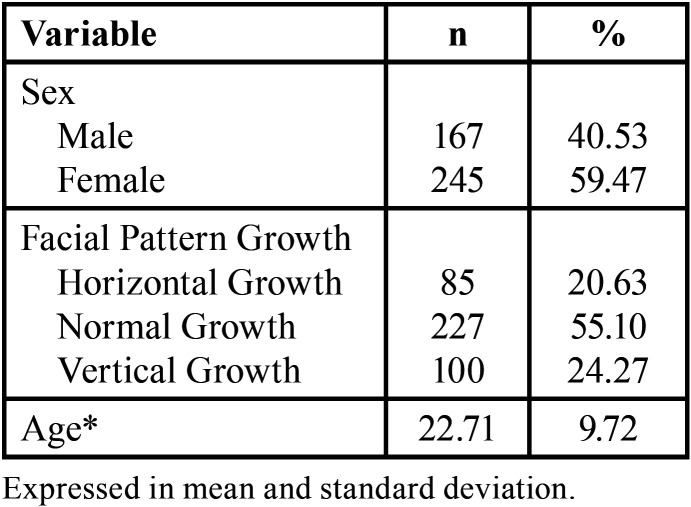
Characteristics of the sample (n=412).

The results showed that the first and second premolars, and maxillary first molar of the groups with horizontal and normal growth presented greater and significantly different angulations in relation to the palatal plane than the group with vertical growth (*p*<0.05). In the case of the maxillary second molar, the angulation was greater for the group with horizontal growth and statistically different from the other groups (*p*<0.05) (Table 3). Regarding the angulation of the maxillary teeth with respect to the occlusal plane, it was observed that the horizontal group presented a greater angulation for the premolars and first molar with respect to the other two groups (*p*<0.05), while for the groups with normal and vertical growth, these differences were not significant. In addition, the second molar presented similar angulations in the three groups (*p*>0.05) (Table 3). The angulations of all mandibular teeth with respect to the mandibular plane were significantly higher for the group with horizontal growth, followed by the group with normal and vertical growth (*p*<0.05) (Table 3). On the other hand, it was observed that when the angulation of the mandibular posterior teeth with respect to the occlusal plane was analyzed, the first and second premolars had similar angulations for the group with horizontal and normal growth, while the angulations for both molars were statistically different between the three groups (*p*>0.05) (Table 3).

**Table 3:**
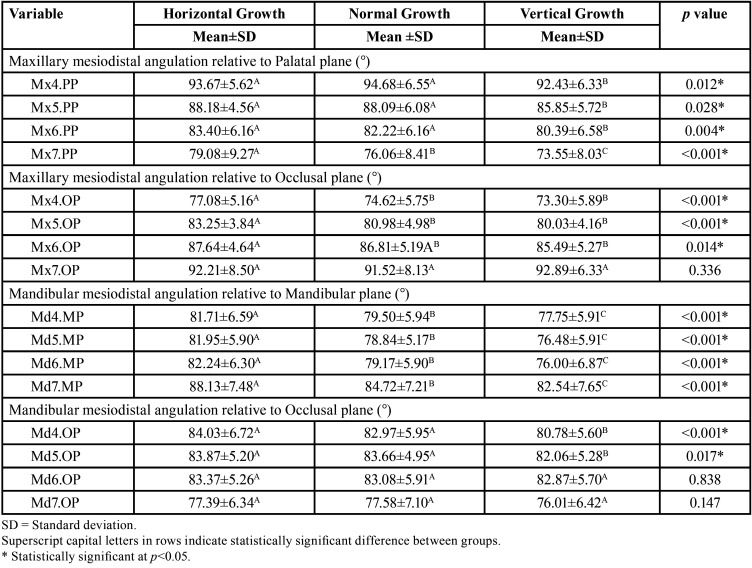
Comparison of all cephalometric magnitudes according to the skeletal growth patterns.

## Discussion

Facial growth patterns have been extensively studied and considerations regarding diagnosis and orthodontic treatment mechanics have been established (23-25). These considerations are mainly related to the rotation tendencies of the jaws and the magnitudes of forces applied with orthodontic appliances or intermaxillary elastics (26,27).

The subjects involved in this study were adult orthodontic patients, considering the stability and maturity of facial characteristics. Upper and lower facial heigh are reached on average at the age of 12 years for women and 15 for men (28).

In this study, the Björk-Jarabak analysis was used to determine the facial skeletal growth pattern of the subjects. This method has proven to be valid for this purpose in various populations, since it is established from the total relationship of the polygon formed by the saddle, articular and goniac angles (29-32). Other strategies such as the use of the angle of the mandibular plane seem not to be adequate for the determination of the facial pattern, due to the high variability (33).

This study evaluated the mesiodistal angulation of posterior teeth in orthodontic patients with different facial growth patterns. The results showed that the pattern with normal growth was the most frequent (55.1%), followed by the vertical growth pattern (24.27%). These results are similar to those reported by Mohammadi Shayan *et al*., who found a distribution of the normal facial pattern of 47.2%, followed by the long facial pattern of 41.4% and the short facial pattern of 11.5% in an Iranian population (34). Magnani *et al*., reported that the most frequent facial pattern in a black Brazilian population was the mesofacial pattern in women (74%), while in men the mesofacial (47%) and dolichofacial (53%) patterns were more balanced (35). On the other hand, Amatya *et al*., found that the hypodivergent facial growth pattern was the most frequent (71.1%), while normodivergent growth was 18.3% in a Nepali population with class I malocclusion (36).

Differences in the angulations of the teeth in the mesiodistal direction in different skeletal patterns have been previously reported (10,20,21). In general, the results of this investigation showed that the average angulation of the teeth decreases from the first premolar to the second molar, likewise it decreases as the growth becomes more vertical. In fact, the group with vertical growth presented minor angulations in the four maxillary and mandibular teeth with respect to the palatal plane, occlusal plane, and mandibular plane.

Nielsen described that subjects with an increase in the lower facial height present a more posterior growth pattern of the mandibular condyle, consequently the expression of growth at the level of the chin is more vertical associated with a vertical pattern of eruption of the posterior teeth and retroclined anterior teeth. On the other hand, in subjects with more horizontal growth, the eruptive pattern presents a considerably mesial migration (10).

Thus, the direction of the skeletal growth pattern appears to be directly associated with the eruptive pattern of the teeth. As previously described, the horizontal facial planes such as the palatal plane and the mandibular plane tend to be more divergent in subjects with vertical growth and increased lower facial height, while in those with more horizontal growth the planes are more parallel to each other (33,37). This characteristic could explain why the means of the angulations of the teeth with respect to these reference planes vary from smaller angulations in the case of subjects with vertical growth when compared to the values of greater angulations in subjects with horizontal growth.

In patients with vertical growth, the verticalization of the teeth can be the consequence of a dental compensation. Thus verticalization can be present with the aim of closing the open bite (38,39). Badiee *et al*., reported that in patients with a vertical growth pattern, the posterior teeth presented forward inclinations; while in those patients with horizontal growth, the inclinations were backwards. Additionally, they reported an increase in the angulation of the maxillary and mandibular teeth in relation to the palatal and mandibular plane in patients with vertical growth, while the angulations decreased in relation to the occlusal plane (20). These differences in the results with those reported in this study may be due to the fact that in the previous studies the inclinations of the teeth were evaluated in subjects with open bite, normal bite and deep bite, while in the present investigation, this variable was not included in the model, because no marked differences were observed in the studied sample that would allow the appropriate classification for its study.

Janson *et al*., compared the angulations of the posterior teeth in patients with open bite and normal occlusion, their results showed that the first and second maxillary and mandibular premolars of the subjects with open bite presented a greater mesial angulation with respect to the occlusal plane than a difference from the subjects with normal occlusion, while the maxillary and mandibular first and second molars presented greater distal inclinations with respect to the palatal and mandibular plane (21). When comparing the mean values of the angulations of the teeth in the group with normal occlusion with respect to the palatal and mandibular plane, Janson *et al*. results were similar to those reported in this investigation.

Further studies are necessary to establish patterns of relationship between the characteristics of the position of the teeth and facial growth patterns, considerations on buccolingual angulations, overjet, overbite and curve of Spee must be taken into account. As well as the study of dental compensation in patients with ortho-surgical requirement.

## Conclusions

The results of the present investigation showed that in relation to the palatal plane, the first and second premolars presented greater angulations in subjects with horizontal and normal growth than in those with vertical growth. The angulations of all mandibular teeth with respect to the mandibular plane were significantly higher for the group with horizontal growth, followed by the group with normal and vertical growth.
